# Differential expression and regulation of prohibitin during curcumin-induced apoptosis of immortalized human epidermal HaCaT cells

**DOI:** 10.3892/ijmm.2014.1621

**Published:** 2014-01-09

**Authors:** HAI-BO YANG, WEI SONG, LAN-YING CHEN, QI-FU LI, SONG-LIN SHI, HAI-YAN KONG, PU CHEN

**Affiliations:** 1 School of Life Sciences and Engineering, Henan University of Urban Construction, Pingdingshan, Henan 467044, P.R. China; 2 Medical College of Xiamen University/Cancer Research Center of Xiamen University, Xiamen, Fujian 361102, P.R. China

**Keywords:** prohibitin, nuclear matrix, curcumin, cell apoptosis, differential expression

## Abstract

Prohibitin (PHB), also known as inhibin, is important in cell proliferation, differentiation and apoptosis. This protein localizes to the inner membrane of mitochondria, where it acts as a chaperone protein, and is also found in the nucleus, where it negatively regulates transcription. The tumor-suppressive role of PHB in cell proliferation appears to be contradictory. In this study, we investigated the existence, localization and alterations in the expression of PHB in the whole cell and nuclear matrix and analyzed its co-localization with the expression products of related genes. The western blot analysis results revealed that PHB exists in the composition of nuclear matrix proteins and that the expression level of PHB is significantly increased in the whole cell and markedly decreased in the nuclear matrix after curcumin (1,7-bis(4-hydroxy-3-methoxyphenyl)-1,6-heptadiene-3,5-dione) treatment. The laser confocal scanning microscope results demonstrated the co-localization of PHB with p53, c-Myc, Bax, and Fas in HaCaT cells, and this co-localization region was transferred as a result of curcumin treatment. In addition, the results of the GST pull-down assay demonstrated the direct interaction of PHB with p53, c-Myc and Bax but not Fas *in vitro*. Results of the present study confirmed that the expression and distribution of PHB, which is a nuclear matrix protein, affect the apoptosis of HaCaT cells and its co-localization with specific gene products connected with cell apoptosis.

## Introduction

Curcumin (1,7-bis(4-hydroxy-3-methoxyphenyl)-1,6-heptadiene-3,5-dione) is the major yellow pigment extracted from turmeric, which is a commonly used spice derived from the rhizome of the *Curcuma longa* plant (1). An increasing number of studies have recently supported its use in cancer prevention therapy due to its anti-proliferative and anti-carcinogenic properties (2). Curcumin not only suppresses carcinogenesis of the skin, stomach, colon and breast *in vivo* but also inhibits the growth of a wide variety of tumor cells *in vitro* (3,4).

Prohibitin (PHB), also known as inhibin, is widely distributed in bacteria, plants, yeast, protozoa and mammals and is important in cell proliferation, differentiation and apoptosis (5). This protein localizes to the inner membrane of mitochondria, where it acts as a chaperone protein (6,7), and is found in the nucleus, where it negatively regulates transcription (8). The *Phb1* gene, which is a member of the PHB family, is located beside the tumor-suppressor gene *BRCA1* in the chromosome, rendering *Phb1* highly relevant in breast cancer (9). Findings of a recent study revealed that the aberrant expression of PHB was clearly overexpressed in gastric, liver and uterine cancer, whereas another study showed that PHB is capable of promoting cell apoptosis by interacting with a specific tumor-suppressor protein (10–12). This finding appears to contradict the tumor-suppressive role of PHB. At present, the mechanism underlying its subcellular localization and the mechanisms through which it regulates cell proliferation and differentiation remain to be elucidated.

In the present study, we investigated the existence, localization and alteration of the expression of PHB in HaCaT cells in response to treatment with curcumin. We also examined the interaction between PHB and proteins related to oncogenes and tumor-suppressor genes during curcumin-induced apoptosis. Thus, results of this study provided new insight on the functions and mechanism of action of PHB as an antitumor target during cell apoptosis.

## Materials and methods

### Materials

Immortalized human epidermal HaCaT cells were obtained from the China Center for Type Culture Collection (Wuhan, China). Goat anti-mouse HRP-IgG, goat anti-rabbit HRP-IgG, the mouse anti-human PHB antibody, and the rabbit anti-human p53, Rb, Fas and c-Myc antibodies were all obtained from Santa Cruz Biotechnology, Inc. (Santa Cruz, CA, USA). RPMI-1640 was purchased from Gibco-BRL (Carlsbad, CA, USA) and newborn calf serum was obtained from Hangzhou Sijiqing Biological Engineering Material Co., Ltd. (Hangzhou, China). Curcumin was obtained from the National Institute of the Control Pharmaceutical and Biological Products (NICPBP) (Beijing, China).

### Cell culture and induction

Immortalized human epidermal HaCaT cells (China Center for Type Culture Collection) were cultured in RPMI-1640 medium supplemented with 10% heat-inactivated fetal calf serum, 100 U/ml penicillin, and 100 mg/ml streptomycin (pH 7.2) at 37°C in air with 5% CO_2_. Twenty-four hours after seeding, the HaCaT cells were maintained in RPMI-1640 with 7.5 mg/l curcumin for 72 h to induce differentiation. HaCaT cells cultured in RPMI-1640 medium were used as the control.

### Cell-selective extraction and sample preparation for light microscopy

The cells were selectively extracted as described in a previous study (13). After selective extraction, the cells were prefixed in 2% glutaraldehyde at 4°C for 30 min, and the nuclear matrix-intermediate filament (NM-IF) samples were coverslipped and rinsed in phosphate-buffered saline (PBS) at pH 7.4. The samples were then stained with 0.2% Coomassie Brilliant Blue for 20 min, washed in distilled water, air-dried, clarified by xylene, enveloped in a resin and observed by Olympus BH-2 microscopy.

### Purification of the nuclear matrix protein

HaCaT cells were washed twice with cold PBS and extracted using a cytoskeleton (CSK100) buffer (100 mM NaCl, 3 mM MgCl_2_, 10 mM PIPES, 300 mM sucrose, 0.5% Triton X-100, 1 mM EGTA, and 1 mM PMSF, pH 6.8) for 10 min at 0°C. After centrifugation at 1,000 × g for 5 min, the pellets were washed with cold PBS to remove soluble cytoplasmic proteins and then re-centrifuged and suspended in the digestion buffer CSK50 (identical to CSK100 buffer, except with 50 mM NaCl instead of KCl) containing 400 U/ml DNase I for 30 min at room temperature. Cold ammonium sulfate was added to a final concentration of 0.25 M to precipitate the proteins. After centrifugation at 1,000 × g for 5 min, the pellets were washed with CSK50 buffer and dissolved in lysis buffer [7 M urea, 2 M thiourea, 4% CHAPS, 1.5% Triton X-100 and 1% pharmalyte (pH 3–10; Amersham Biosciences), 65 mM DTT, 40 mM Tris, 5 mg/ml aprotinin, 1 mg/ml leupeptin, 1 mg/ml pepstatin, 2 mM PMSF and 4 mM EDTA]. The sample was sonicated at 0°C for 30 min and centrifuged at 10,000 × g for 1 h. The protein concentrations of the control and treated groups were determined using the Bradford assay, and the proteins were stored at −80°C.

### Western blot analysis

The protein lysates were electrophoretically separated in 12% polyacrylamide gels and transferred onto PVDF membranes. The membranes were incubated in 5% non-fat milk for 1.5 h at room temperature to block any non-specific binding and then incubated with mouse PHB nucleophosmin antibody (1:1,000 dilution; Santa Cruz Biotechnology, Inc.) in TBST for 1 h. The membranes were then washed once with TBST for 10 min, incubated with horseradish peroxidase-conjugated goat anti-mouse IgG (1:10,000 dilution; Santa Cruz Biotechnology, Inc.) as the secondary antibody for 1 h at room temperature, and washed three times with TBST for 30 min. Immunoreactive bands were identified using an enhanced chemiluminescence (ECL) detection system (Pierce Biotechnology, Inc., Rockford, IL, USA). Samples incubated with 5% non-fat milk instead of the primary antibodies were used as negative controls. In addition, β-actin was used as an internal control.

### Sample preparation for fluorescent microscopy

The NM-IF samples on the cover slip were prefixed in 4% paraformaldehyde at 4°C for 10 min, rinsed twice in TPBS (containing 0.5% Triton X-100) for 10 min, and blocked with 5% BSA at room temperature for 1 h. The samples were incubated with mouse anti-PHB antibody (1:300 dilution) at room temperature for 30 min, incubated overnight at 4°C, and then washed three times with TPBS. The cells were then incubated with goat anti-mouse secondary antibody labeled with the fluorescent dye FITC, washed in water, and air-dried. Then, 90% glycerol in PBS was applied, and the cells were observed by fluorescence microscopy. All of the steps after incubation with the secondary antibody were performed in the dark, and samples incubated with 5% BSA instead of the primary antibody were used as the negative control.

### Sample preparation for LSCM

The cells on the cover slips in the curcumin and control groups were rinsed three times in PBS for 15 min and submerged in 0.1 M TBS (containing 0.5% Triton X-100) for 20 min at room temperature. The cells were fixed in 4% paraformaldehyde at pH 7.2 for 10 min, washed three times with PBS (pH 7.2) for 15 min, blocked with 5% BSA at room temperature for 1 h, and then incubated with dual primary antibodies at room temperature for 30 min and at 4°C overnight. The dual sets of primary antibodies were: PHB (1:50)/Fas (1:30), PHB (1:50)/c-Myc (1:30), PHB (1:50)/P53 (1:30), PHB (1:50)/Bax (1:30), and PHB (1:50)/Bcl-2 (1:30). After washing with TBS, the cells were incubated with different secondary antibody sets (goat anti-mouse and goat anti-rabbit, both diluted at 1:200) and incubated at room temperature for 3 h in the dark. The cells were then washed three times with PBS for 30 min, enveloped with an anti-fluorescence quencher after drying, blocked with nail polish, and observed under TSC-SP2-MP LSCM.

### GST pull-down assay

The samples were inoculated with non-carrier bacterial plasmids, and the constructed prokaryotic expressive strains were induced through soluble expression. The GST protein and GST combined protein were refined using glutathione sepharose 4B following the procedure described above. RIPA lysis buffer was used to dissolve the HaCaT cells, and the cells were centrifuged at 12,000 × g and 4°C for 5 min. The centrifuged cell lysates were incubated with GST and GST-PHB combined with beads at 4°C for 1 h in a silent mixer. The cells were then washed three times using the GST washing buffer. Then, 5X SDS loading buffer was added for SDS-PAGE, and the same amount was added to the cell lysates as described previously (16). The interaction between the prey and bait protein was verified by western blot analysis.

## Results

### Detection of PHB in the whole cells and nuclear matrix by western blot analysis

**Figure 1:**
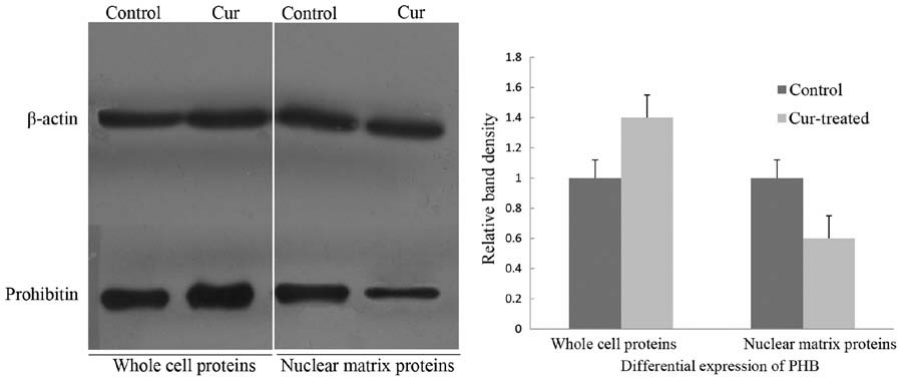
Expression of prohibitin (PHB) protein in HaCaT cells detected at whole cell and nuclear matrix proteins by western immunoblotting. Expression of PHB was found to be upregulated at whole cell and downregulated at nuclear matrix after curcumin (1,7-bis(4-hydroxy-3-methoxyphenyl)-1,6-heptadiene-3,5-dione) treatment.

To verify the aberrant changes in PHB, western immunoblotting was employed to confirm the expression levels of PHB in the whole cells and nuclear matrix prior to and following curcumin treatment, and the intensities of the protein bands were densitometrically quantified as described by Sheffield (14). The expression level of PHB in whole cells after exposure to curcumin for 48 h was increased, whereas the level of the PHB was significantly decreased in the nuclear matrix protein (Fig. 1). Thus, the PHB expression in whole cells and the nuclear matrix of HaCaT cells was inversely correlated.

### Localization and expression of PHB in HaCaT cells

**Figure 2:**
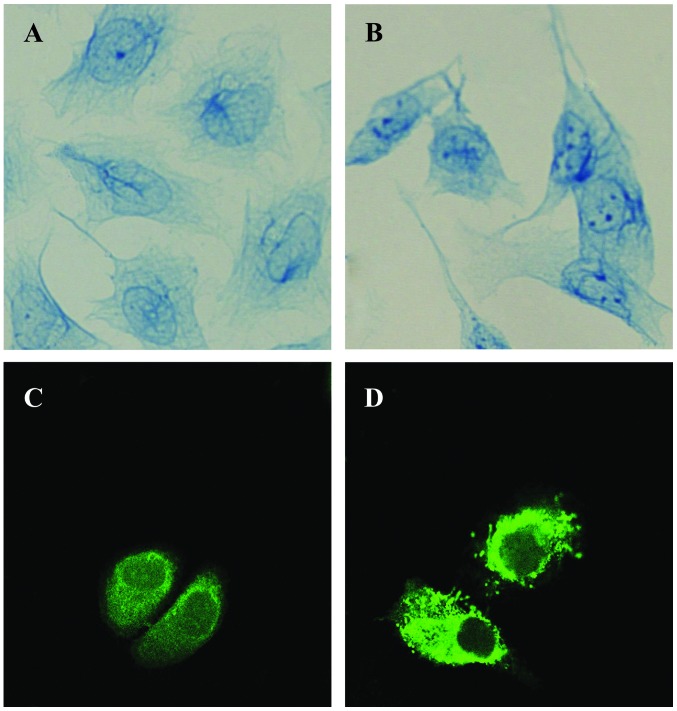
Effects of curcumin (1,7-bis(4-hydroxy-3-methoxyphenyl)-1,6-heptadiene-3,5-dione) treatment on the localization of differentially expressed nuclear matrix proteins in the nuclear matrix-intermediate filament (NM-IF) system. (A) Light microscopy observation of NM-IF system in HaCaT cells, stained by Coomassie Brilliant Blue. (B) Light microscopy observation of the NM-IF system following curcumin treatment in HaCaT cells, stained by Coomassie Brilliant Blue. (C) Immunofluorescence staining of prohibitin (PHB) in HaCaT cells. (D) Immunofluorescence staining of PHB following curcumin treatment in HaCaT cells.

The light microscopy observations showed that the IF in HaCaT cells was sparse and distributed irregularly in the nucleus. In the curcumin-treated cells, the entire framework became more widespread, whereas the NM-IF results showed characteristics of uniform distribution. The evenly stained intermediate filaments spread from the region around the nucleus to the edge of the cell to form a uniform network throughout the cytoplasm (Fig. 2A and B).

The immunofluoresence analysis revealed the localization and expression of PHB in HaCaT cells; the PHB protein was labeled with FITC (green). The results revealed that PHB was distributed throughout the cell. Its signal was strong in the cytoplasm but weak in the nucleus, where it was distributed as small particles, especially at the edge of nuclei; in particular, the PHB signal was relatively strong in the nearby nuclear membrane region. PHB was also evenly distributed in the cytoplasmic region (Fig. 2C). The analysis of curcumin-treated cells through microscopy showed that the distribution and expression of PHB changed significantly. The brightness of the fluorescence in the nucleus of curcumin-treated HaCaT cells was obviously weakened, whereas the brightness of the fluorescence of the cytoplasm of these cells was strengthened. This finding indicates that PHB tended to move from the nuclear matrix to the lamina and cytoplasm (Fig. 2D).

### LSCM analysis of the co-localization of PHB with oncogenes and tumor-suppressor genes during curcumin-induced apoptosis

The localization of PHB and its related proteins, c-Myc, Bax, p53 and Fas, was observed by LSCM. PHB was labeled with FITC (green), and the other proteins were labeled with TRITC (red). The co-localization fluorescence was yellow or orange, i.e., the combination of the two different colors.

In HaCaT cells, PHB was distributed almost throughout the entire cell. Compared with its distribution in the cytoplasm, the amount and distribution of PHB in the karyoplasm was lower and uneven, respectively. In response to curcumin treatment, the level of PHB was enhanced and widely distributed in the cytoplasm of HaCaT cells and decreased or even absent in the karyoplasm.

### Co-localization of PHB with c-Myc in HaCaT cells

**Figure 3:**
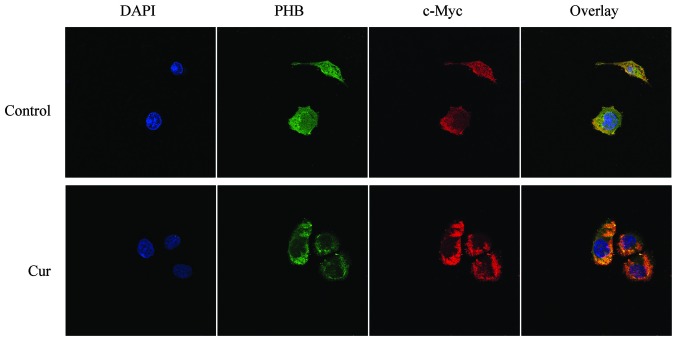
The co-localization between prohibitin (PHB) and c-Myc in HaCaT cells before and after treatment with curcumin (1,7-bis(4-hydroxy-3-methoxyphenyl)-1,6-heptadiene-3,5-dione). These results were observed by LSCM, PHB was labeled with FITC (green), c-Myc was labeled with TRITC (red). The co-localization fluorescence was yellow or orange when the two different colors overlapped.

Fluorescence microscopy revealed that the nuclei of HaCaT cells stained with DAPI were blue in the control and curcumin-treated group. The c-Myc protein, which was labeled by TRITC, emitted a red fluorescence and was mainly distributed in the cytoplasmic region. By contrast, the distribution of the green fluorescence showed that PHB was distributed near the nuclear membrane region, whereas other locations exhibited weaker fluorescence. The yellow in the overlaid fluorescence image indicated that the co-localization of PHB and c-Myc was found only slightly in the nucleus edge region but at a higher amount in the nucleolus. However, in the curcumin-treated HaCaT cells, both the green fluorescence in the cytoplasm and the red color representing c-Myc were strengthened. The co-localization between the two proteins in the curcumin-treated cells was enhanced in the cytoplasm, a result that most likely demonstrates that the co-localization region of the two proteins was transferred from the nucleus to the cytoplasm in response to curcumin treatment (Fig. 3).

### Co-localization of PHB with p53 in HaCaT cells

**Figure 4:**
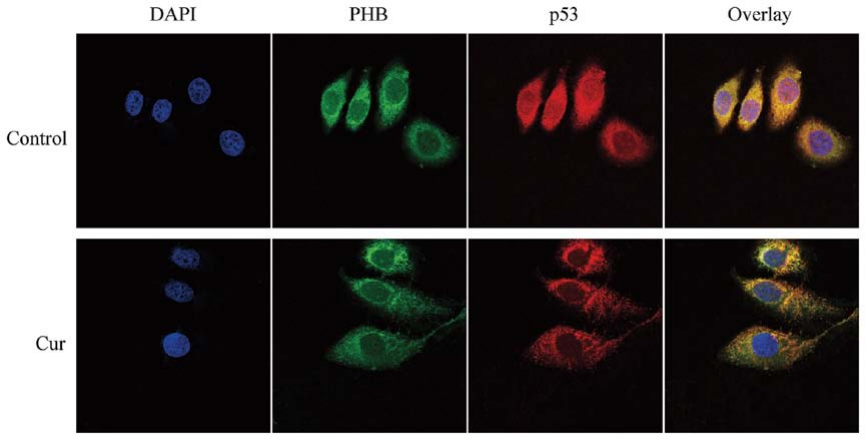
The co-localization between prohibitin (PHB) and p53 in HaCaT cells before and after treatment with curcumin (1,7-bis(4-hydroxy-3-methoxyphenyl)-1,6-heptadiene-3,5-dione). These results were observed by LSCM, PHB was labeled with FITC (green) and p53 was labeled with TRITC (red). The co-localization fluorescence was yellow or orange when the two different colors overlapped.

In the control HaCaT cells, the green fluorescence representing PHB was distributed throughout the cell, although it was higher near the nuclear membrane in the cytoplasmic region and weaker in the other cell regions. p53, which was labeled with TRITC, exhibited red fluorescence that was distributed throughout the cell, but the fluorescence intensity was higher in the cytoplasm and nucleolus. The yellow fluorescence (overlaid signals) indicated that the co-localization between PHB and p53 was high in the nuclear membrane region. The curcumin-treated HaCaT cells exhibited weaker fluorescence throughout the entire cell. The green color was scattered in the cytoplasm and decreased in the nucleus; however, the nucleolus exhibited green fluorescence. In addition, p53 was distributed in the cytoplasmic region and exhibited a weaker fluorescence in the nucleolus of these curcumin-treated cells. The overlapped yellow fluorescent color demonstrated that PHB and p53 are co-localized in the nuclear membrane region, particularly in the cytoplasmic region. In addition, the co-localization in the nucleolus was markedly lower compared with that found in the control cells. The abovementioned evidence suggests that the co-localization region of the two proteins moved from the nucleus to the cytoplasmic region in response to curcumin treatment (Fig. 4).

### Co-localization of PHB with Fas in HaCaT cells

**Figure 5:**
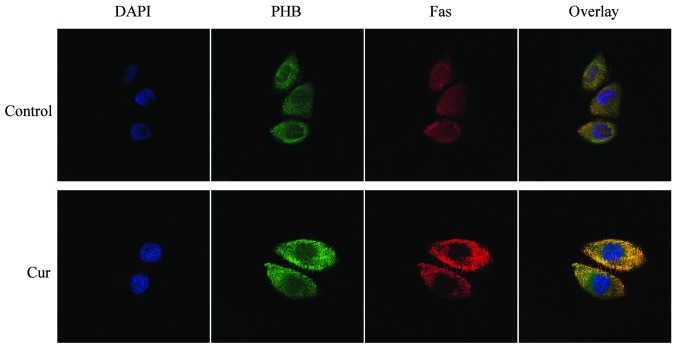
The co-localization between prohibitin (PHB) and Fas in HaCaT cells before and after treatment with curcumin (1,7-bis(4-hydroxy-3-methoxyphenyl)-1,6-heptadiene-3,5-dione). These results were observed by LSCM, PHB was labeled with FITC (green) and Fas was labeled with TRITC (red). The co-localization fluorescence was yellow or orange when the two different colors overlapped.

According to the fluorescent staining results, the red fluorescence, which represented Fas, was well distributed throughout the cell. Compared with the fluorescence intensity in the nucleus, the intensity increased near the nuclear membrane region. The yellow fluorescence clearly revealed the co-localization of PHB and Fas in the nucleolus and around the nuclear membrane. The green fluorescence, which represented PHB, was weaker in the HaCaT cell nucleus following treatment with curcumin and was increased in the cytoplasm. In addition, after curcumin treatment, Fas was well distributed in the cytoplasm and was slightly weaker in the nucleus, although its fluorescence remained strong. The co-localization of PHB and Fas after curcumin treatment suggests that the two proteins were clearly co-localized around the cytoplasm and the cytomembrane, although the co-localization relationship in the nucleus was weakened or completely absent. This finding suggests that the PHB and Fas co-localization region shifted from the nucleus to the cytoplasm in response to curcumin treatment (Fig. 5).

### Co-localization of PHB with Bax in HaCaT cells

**Figure 6:**
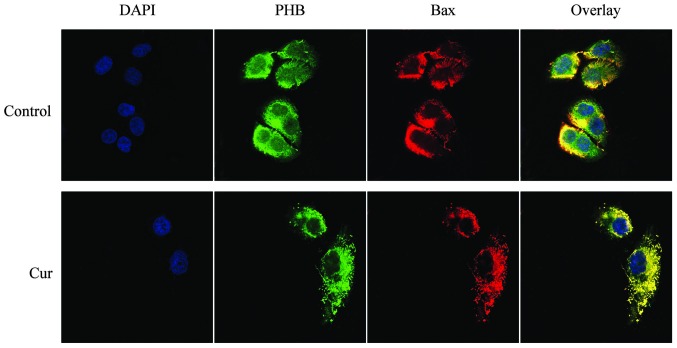
The co-localization between prohibitin (PHB) and Bax in HaCaT cells before and after treatment with curcumin (1,7-bis(4-hydroxy-3-methoxyphenyl)-1,6-heptadiene-3,5-dione). These results were observed by LSCM, PHB was labeled with FITC (green) and Bax was labeled with TRITC (red). The co-localization fluorescence was yellow or orange when the two different colors overlapped.

Bax was labeled with TRITC and thus exhibited a red fluorescence. The LSCM results showed that Bax was distributed throughout the cell, although the fluorescence intensity was weaker in the nucleus but stronger around the nucleolus. Similarly, PHB exhibited green fluorescence that was well distributed in the nucleus, although the fluorescence intensity was mostly clustered near the nucleolus. The overlaid yellow fluorescence of the two proteins was strongly co-localized near the nucleus and less co-localized in the nucleolus. After treatment with curcumin, the level of PHB decreased significantly in the nucleus, and the green fluorescence was clearly enhanced in the cytoplasm. Following curcumin treatment, the red fluorescence, which represents Bax, decreased significantly or even disappeared in the nucleus and was significantly enhanced in the cytoplasm. The yellow fluorescence showed that the two proteins were clearly co-localized in the cytoplasm and not co-localized in the nucleus. This finding demonstrates that the co-localization of PHB and Bax shifted from the nucleus to the cytoplasm in response to curcumin treatment (Fig. 6).

### Interaction of PHB with p53, c-Myc, Bax and Fas in HaCaT cells

**Figure 7:**
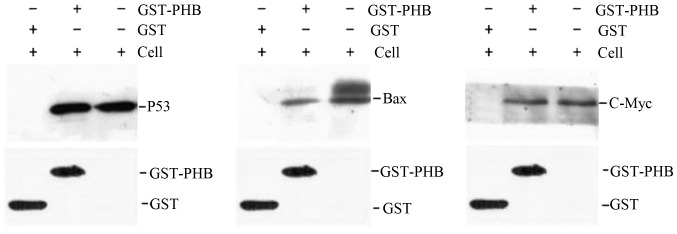
Interactions between prohibitin (PHB) and the related tumor proteins (c-Myc, Bax and p53). GST pull-down assay was employed to verify the interaction between PHB and c-Myc, Bax and p53 in HaCaT cells.

To verify the interaction between PHB and the oncogene proteins in HaCaT cells, a GST pull-down assay was employed. GST-PHB and GST were expressed in bacteria and purified to near homogeneity. After the recombinant proteins GST-PHB and GST were incubated with the HaCaT cell lysate supernatant, the western blotting results suggested the existence of a direct interaction between GST-PHB and p53 *in vitro*, although no interaction was identified between GST and p53 (Fig. 7). Identical results were obtained for the analysis of GST-PHB with c-Myc and Bax, and no significant interaction was detected between GST-PHB and Fas (data not shown). As a result, we suggest that PHB directly interacts with p53, c-Myc and Bax, and these results are consistent with the cellular co-localization results (Fig. 7).

## Discussion

### Expression and subcellular localization changes of PHB

The PHB protein is encoded by the PHB gene, which is related to cell apoptosis, and is found to be diffused throughout all eukaryotic cells, which means that it is highly conserved in evolution. Results of a previous study have shown that PHB is upregulated in tumor cells compared with normal cells (15). Results of the present study show that PHB was upregulated in whole cells and markedly downregulated in the nuclear matrix. Additionally, we found that PHB is translocated from the nuclei to the nuclear membrane and cytoplasm after curcumin treatment. These results indicate that PHB is found not only in the mitochondria of the cytoplasm but also in the nuclear matrix of HaCaT cells and that its decreased nuclear expression and localization is associated with canceration and reversion. These results are consistent with those of our previous study, which suggests that PHB is capable of being downregulated and transported from the cytoplasm to the nucleus in osteosarcoma MG-63 cells (16). This ability of PHB may be one of the molecular mechanisms associated with its effectiveness in the prevention of cancer. There are conflicting experimental results regarding the manner in which a microinjection of PHB mRNA blocks cell proliferation (17) and in the manner in wihch PHB functions as a tumor-suppressor protein and arrests G1-S cell transition through a process involving the repression of E2F-mediated transcription (18). We hypothesized that PHB has different functions in different cell lines, that its expression levels are related to the tumor clinical stage, and that it may act as a molecular switch that controls cell proliferation.

### Alteration of the co-localization and interaction between PHB and other oncogenes and tumor-suppressor genes

The immunofluorescence microscopy and LSCM results revealed that PHB co-localizes with the products of p53, c-Myc, Bax and Fas genes in HaCaT cells and that the location of this co-localization is altered by curcumin treatment. In addition, the GST pull-down assay showed that PHB directly interacts with p53, c-Myc and Bax in HaCaT cells.

The tumor-suppressor gene p53 is a type of specific transcription factor and is highly conserved in evolution, similar to PHB. p53 is important in the inhibition of the cell cycle, the promotion of cell apoptosis and aging (19). DNA damage cannot be repaired in time if the p53 gene is in an inactive state, and the accumulation of DNA damage contributes to the conversion of a cell into a tumor cell. Our LSCM results indicate that the co-localization of p53 and PHB is unevenly distributed, mainly in the nuclear region. However, the co-localization location of the two proteins was altered following treatment with curcumin. This phenomenon shows that the interaction between the two proteins may regulate cell apoptosis, and these results were also verified through the GST pull-down experiment.

Additionally, the c-Myc oncogene encodes the c-Myc protein. The c-Myc protein is a type of transcription factor, and its expression has been found to be increased in most human tumor cells. In addition, c-Myc has the dual effect of promoting cell proliferation and apoptosis (20). Findings of a previous study have shown that c-Myc is important in mammalian cell apoptosis (21). Therefore, c-Myc is the main factor that regulates cell proliferation, differentiation, apoptosis, canceration and metastasis. Our LSCM results revealed that the PHB and c-Myc co-localization is found in the nucleolus and near the nucleus in control cells and is mainly found in the cytoplasm following curcumin treatment. This change in the co-localization location suggests that there is a close relationship between the interaction of the two proteins and cell apoptosis, as was confirmed by the GST pull-down results.

Bax, which is located in the cytoplasm, belongs to the Bcl-2 family and is another factor that controls cell apoptosis. Homodimers of Bax or the combination of Bax and Bcl-2 are capable of inducing cell apoptosis (22). However, the Bax/Bac-2 dipolymer may exhibit a rivalry with Bcl-2 in the inhibition of apoptosis. These proteins regulate cell apoptosis mainly by controlling the transfer of Bax to the mitochondria (23). The LSCM results obtained in this study suggest that the location of the co-localization between Bax and PHB is altered from the nucleus to the cytoplasm following treatment with curcumin. This finding suggests the hypothesis that the expression of Bax is increased via its interaction with PHB, which prevents Bax from translocating to the mitochondrion and promotes the induction of the apoptosis pathway. The findings obtained in our study suggest that the interaction of Bax with PHB after PHB is transported out of the nucleus leads to the activation of an apoptosis signal factor, which contributes to the death of HaCaT cells.

The interaction of Fas with its natural ligand FasL activates the apoptosis signal for transmembrane delivery and is thus termed the ‘dead ligand’. The Fas protein is expressed in numerous tumor cells, and the Fas and FasL compounds may activate the cascade reaction of the caspase family, thereby inducing the apoptosis of target cells (24,25). The LSCM results of the present study reveal that Fas is found mainly in the cytoplasm and the nucleus and that its co-localization with PHB is mainly found in the nucleolus and cell cytoplasm. Following treatment with curcumin, the location of the co-localization of the two proteins was altered in the cytoplasm, which demonstrates the relationship between the interaction of the two proteins and HaCaT cell apoptosis. However, these results were not confirmed through our GST pull-down experiment. Therefore, additional investigation on the Fas protein is required as there are no studies on the relationship between the interaction of PHB and Fas and cell apoptosis.

In this study, we confirmed the activity of PHB as a significant regulatory factor in the proliferation and apoptosis of HaCaT cells. This conclusion was based on the findings after curcumin treatment that its expression was decreased in the nucleus and increased in the whole cell and that PHB is co-localized and translocated with oncogene proteins and tumor-suppressor proteins. We provide evidence for additional investigation to be conducted into the function of PHB during the proliferation of human epidermal HaCaT cells. Additionally, the described interactions of PHB with the proteins encoded by oncogenes and tumor-suppressor genes offer new insight into the mechanism underlying the development of canceration and its reversion.
